# Qualitative Analysis of Palliative Care for Pediatric Patients With Cancer at Bugando Medical Center: An Evaluation of Barriers to Providing End-of-Life Care in a Resource-Limited Setting

**DOI:** 10.1200/JGO.17.00047

**Published:** 2018-01-16

**Authors:** B. Emily Esmaili, Kearsley A. Stewart, Nestory A. Masalu, Kristin M. Schroeder

**Affiliations:** **B. Emily Esmaili** and **Kearsley A. Stewart**, Duke University; **Kristin M. Schroeder**, Duke University Medical Center, Durham, NC; and **Nestory A. Masalu**, Bugando Medical Center, Mwanza, Tanzania.

## Abstract

**Purpose:**

Palliative care remains an urgent, neglected need in the developing world. Global disparities in end-of-life care for children, such as those with advanced cancers, result from barriers that are complex and largely unstudied. This study describes these barriers at Bugando Medical Center, one of three consultant hospitals in Tanzania, to identify areas for palliative care development suitable to this context.

**Methods:**

In-depth interviews were conducted with 20 caregivers of pediatric patients with cancer and 14 hospital staff involved in pediatric end-of-life care. This was combined with 1 month of participant observation through direct clinical care of terminally ill pediatric patients.

**Results:**

Data from interviews as well as participant observation revealed several barriers to palliative care: financial, infrastructure, knowledge and cultural (including perceptions of pediatric pain), and communication challenges. Although this study focused on barriers, what also emerged were the unique advantages of end-of-life care in this setting, including community cohesiveness and strong faith background.

**Conclusion:**

This study provides a unique but focused description of barriers to palliative care common in a low-resource setting, extending beyond resource needs. This multidisciplinary qualitative approach combined interviews with participant observation, providing a deeper understanding of the logistical and cultural challenges in this setting. This new understanding will inform the design of more effective—and more appropriate—palliative care policies for young patients with cancer in the developing world.

## INTRODUCTION

Despite successes in reducing the global burden of childhood cancers, the rate of new cancers is rapidly increasing in low- and middle-income countries (LMICs).^1^ Today, 80% of the global cancer burden rests on countries with only 5% of available medical resources.^2^ Most childhood cancers occur in settings where late diagnosis and inconsistent access to care compound the challenges of significant resource limitations.^3^ The majority of cancer deaths—upward of two thirds—occur in LMICs.^4^ In resource-poor settings worldwide, many children with cancer are left with terminal diagnoses and unnecessary suffering.^5,6^

Global disparities in end-of-life care largely mirror disparities in cancer care.^7,8^ In places where curative treatment is not an option for children with cancer, palliative care could offer a realistic, compassionate treatment plan. The WHO asserts that palliative care is an essential part of the minimum health care package.^9^ However, palliative care for children in the developing world remains largely absent; an estimated 1.2 million children need palliative care globally. The majority (98%) live in LMICs.^10,11^ And just as pediatric cancer care faces complex challenges in LMICs, pediatric palliative care comes with its own obstacles—barriers that are multidimensional, layered, and inadequately understood. Most literature on pediatric palliative care in low-resource settings focuses on financial and infrastructure barriers^11,12^; little has been written on deeper, more complex barriers or on potential solutions. Additional multidisciplinary research is needed to realize feasible solutions and ultimately to inform policy changes for successful palliative care implementation.

To better understand these complex barriers and inform solutions in LMIC settings, this study explored end-of-life care for children at Bugando Medical Center (BMC) in Mwanza, Tanzania. BMC is a 900-bed referral hospital serving a catchment area of > 15 million people. It is one of three hospitals in the country that treat children with cancer. Historically, 2-year survival rates at BMC were 20%, with 52% of cancer diagnoses considered palliative at presentation, because of late presentation and limited diagnostic and therapeutic resources (K. Schroeder, personal communication, June 15, 2016).^13^ No national health insurance plan exists in Tanzania, and 67.9% of the population lives below the poverty line.^14,15^ With the average cost of a cancer diagnosis and treatment at $560 per patient,^16^ most caregivers cannot afford life-saving interventions for patients, resulting in a large number of terminally ill children in Tanzania.

The aim of this study was to use multidisciplinary methods to better describe the barriers to palliative care for pediatric patients with cancer in Tanzania. In addition, this study aimed to identify strengths that could help overcome these complex barriers, to inform the development of culturally appropriate palliative care policies for children in Tanzania and in similar settings globally.

## METHODS

Ethics approval was obtained through Duke University Institutional Review Board (PROD0560), Bugando Medical Center/Catholic University of Health and Allied Sciences Ethics Review Committee, and the Tanzanian National Institute for Medical Research (MR/53/100/432; approved September 7, 2016). Data were gathered from two primary sources: semistructured interviews with caregivers and hospital staff, and direct participant observation.^17^ By giving equal weight to both informant groups, this study attempted to acknowledge perspectives of patients and their families alongside those of hospital staff. Participants for caregiver interviews were randomly selected among caregivers of pediatric patients (< 18 years old) presenting with terminal diagnoses. A terminal diagnosis was defined as having no further curative treatment available in Tanzania as designated by the medical team (Table 1). All caregivers who were approached agreed to participate.

**Table 1:**
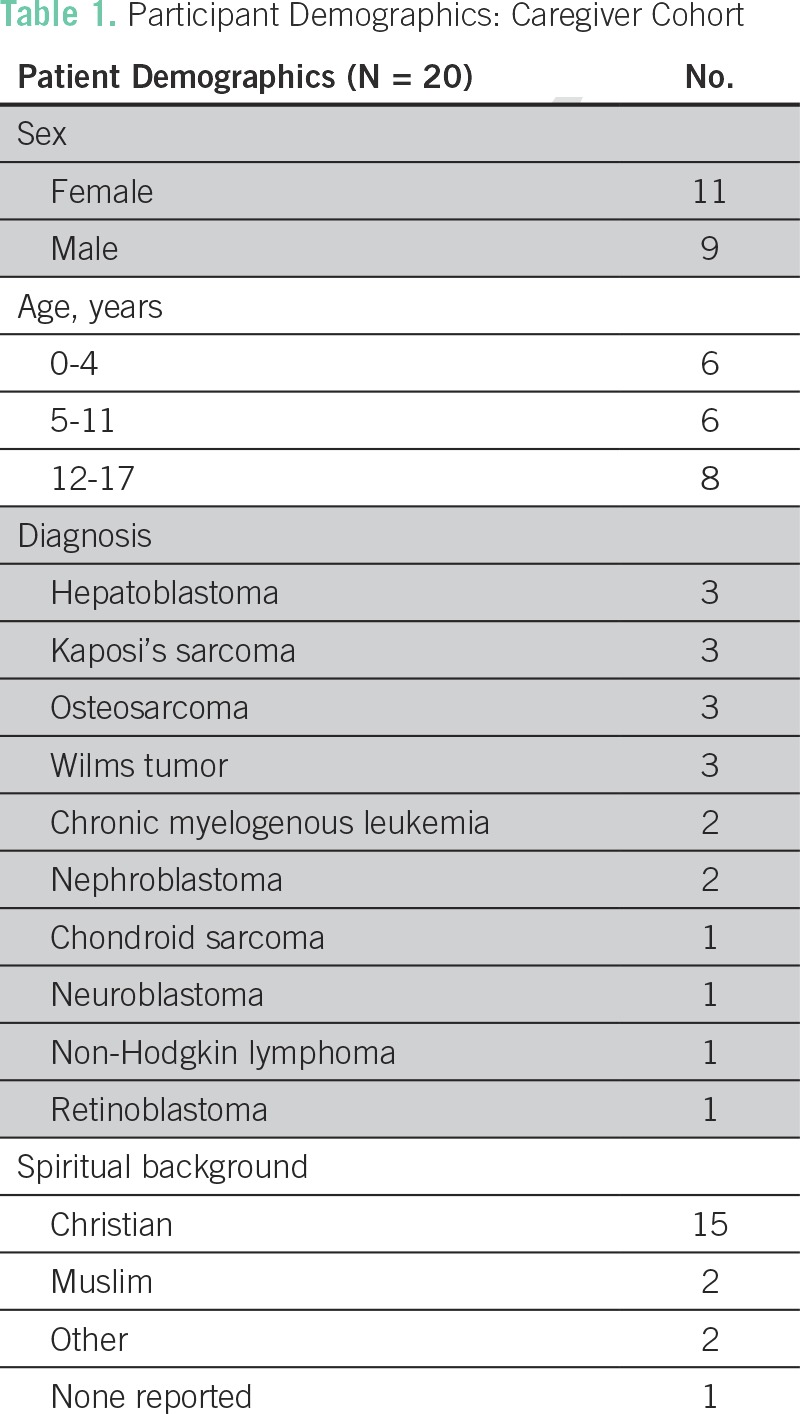
Participant Demographics: Caregiver Cohort

Hospital staff interviews were completed by chaplains, doctors, nurses, and social workers randomly selected from a range of hospital departments involved in the care of children with terminal illness, including oncology, HIV, and intensive care (Table 2). All staff who were approached agreed to participate. All interviews were recorded on a voice recorder, transcribed, and translated into English by two members of the on-site research team.

**Table 2:**
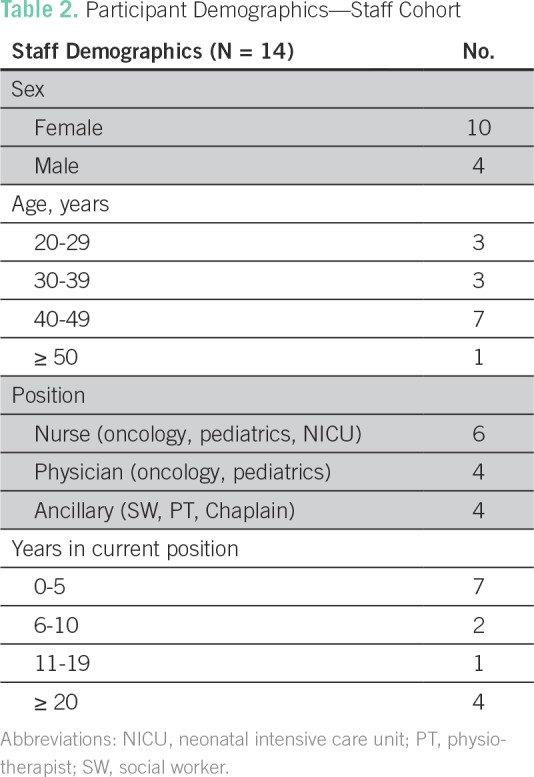
Participant Demographics—Staff Cohort

A context-specific interview questionnaire for caregivers of pediatric patients with cancer and another for hospital staff were developed by the research team, which included a medical anthropologist, sociologist, bioethicist, pediatric oncologist, and pediatric palliative care specialist (Appendix). The semistructured interviews addressed several key issues: staff and caregiver perceptions of end-of-life needs, financial barriers and resource needs, infrastructure barriers, perceptions of pain and knowledge of pain management, and sources of strength in delivering end-of-life care, including faith background. Faith was defined as belief in God or religion or trust in a higher power.^18^ In addition, to assess the prevalence of pain in this sample group, pediatric patients were asked to rate their pain using the Wong-Baker pediatric FACES scale during caregiver interviews.^19^

For a 1-month period, the care of terminally ill pediatric patients was directly observed (B.E.E.) on the oncology ward. Observations were recorded on key issues covered in the questionnaires, including: perceptions of end-of-life needs on the wards; perceptions of pediatric pain; communication between staff, patients, and caregivers; clinical dilemmas created by limited resources; and illustrations of faith as a source of strength. Detailed field notes describing observed experiences were kept daily for a total of 4 weeks.

Results from these two methods were systematically analyzed and coded to extract relevant themes. Following DeCuir-Gunby et al,^20^ the authors developed a preliminary codebook through an interactive, data-driven (inductive) approach and then validated it using a theory-driven (deductive) approach. Supplemental themes were then developed according to Saldana coding schema.

## RESULTS

From May to July 2016, a total of 34 individual in-depth interviews were completed: 20 caregiver and 14 staff interviews. Analysis of coded interviews and participant observation revealed five central themes: financial barriers, infrastructure barriers, knowledge and cultural barriers, communication barriers, and identification of the unique advantages of end-of-life care in this setting (Table 3).

**Table 3:**
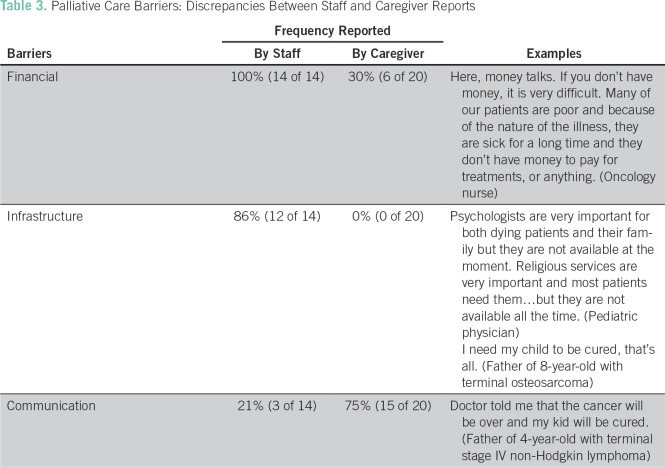
Palliative Care Barriers: Discrepancies Between Staff and Caregiver Reports

### Financial Barriers

Financial needs were the most frequently reported barrier to pediatric end-of life-care. All staff participants (100%) cited resource constraints as a major limitation to the care of terminally ill pediatric patients. Staff reported that patients were often too poor to pay for prescribed medicines, hospital supplies, laboratory tests, or even food, clothes, soap, and hospitalization fees. These resource limitations inhibited the staff’s ability to provide not only basic medical care but also comfort care at the end of life, such as effective pain medication or therapeutic oxygen.

Because we are poor sometimes we have to borrow money. Up to now, we have not paid our house rent. Sometimes it is difficult to bring the child to hospital for treatment. (Mother of 6 year-old with Wilms tumor)

Financial barriers often caused caregivers to demand early discharge in efforts to mitigate costs. As a result, unstable, acutely ill children would leave the hospital when their need for care was the greatest. In addition, the threat of having to pay for postmortem care (if a child died during hospitalization) was observed to be particularly distressing.

### Infrastructure Barriers

Most staff (86%) identified deficiencies in infrastructure or hospital resources, such as consistently available feeding tubes, blood products, and pain medications, that negatively affected their ability to care for terminally ill pediatric patients. Most staff reported using acetaminophen or diclofenac to treat terminal cancer pain in their pediatric patients. Fifty-seven percent also mentioned morphine as a useful treatment, but another 50% reported that it was often out of stock and/or too expensive for families to afford.

Thirty-six percent of staff reported inadequate time to care for terminally ill children as another barrier to care. BMC had no full- or part-time positions for palliative care providers. Several staff expressed interest in dedicating their careers to palliative care; however, no employment opportunities existed at BMC. Staff did identify certain consult services for palliative care, including social work, physiotherapy, and chaplaincy. Fifty percent of staff stated that social workers were the most useful consultants, largely because of their ability to access financial resources, such as donations or payment exemptions. However, staff also reported inconsistent availability of these consult services:

We have [consultants], but it is difficult to get them to come here…. They say there’s nothing to do here, so they don’t like to come. For counseling, most staff don’t do this because they don’t have the time…. Maybe they have the time but they don’t have the heart. (Oncology nurse)

None of the caregivers identified infrastructure barriers to their child’s care; their ultimate wish for curative treatment outweighed any specific wishes for hospital services or programs. If cure was not possible, most wished to go home—largely for financial reasons.

### Knowledge and Culture Barriers

A third thematic barrier that emerged was the stigmatization of death and suffering in Tanzanian culture, which complicated in-hospital palliative care and pain management. According to interview data, most staff reported awareness of palliative care principles, including physical and psycho-spiritual needs of dying children. A few staff reported learning these principles from trainings conducted by visiting specialists, although most reported gaining knowledge through routine clinical experience.

Despite this reported awareness, stigma against dying patients was frequently observed. Pediatric patients with terminal illness were often found segregated or neglected. For example, a 14-year-old with advanced osteosarcoma was hospitalized for weeks while awaiting surgery for disarticulation of her affected leg. As it became clear that this life-saving surgery was not possible, staff slowly backed away from her—spending less time with her on ward rounds, ignoring requests from caregivers for medications or bedpans, and infrequently checking vital signs. One staff respondent validated these observations as follows:

People believe that if [patients] will soon die, they have a stigma and people don’t want to go near them. When they are sent home…no one will feed them or even clean them. Their bed sheets will get wet and they get bedsores. There is this problem with stigma. (Oncology nurse)

Even when hospital resources and infrastructure existed, observations on the wards revealed negative attitudes toward the dying that created another barrier to end-of-life care. Regarding awareness of pediatric pain, all staff stated that most or many of their patients experienced pain, which they identified by excessive crying, irritability, inability to sleep or restlessness, refusal to eat, or vital sign changes such as tachycardia or oxygen desaturations. Although awareness of pain seemed appropriate, the lack of effective treatments, such as morphine, created an additional barrier to pain management.

From the perspective of caregivers, 65% reported that their child had suffered from pain during hospitalization. However, when these same children were asked about their pain during interviews using the Wong-Baker pediatric FACES scale,^19^ only four patients (20%) reported currently having any pain. It was also observed that many patients who showed physical signs of pain denied having pain when asked, which led to under-recognition by staff.

### Communication Barriers

Communication barriers comprised a fourth main theme that recurred throughout the study. Interviewees noted a lack of communication between staff and terminally ill patients. One staff member explained the cultural norms that dictated these communication patterns:

We can never mention dying in our culture, even when that is the case. No one will ever say it. (Social worker)

Many caregivers reported feeling ignored, avoided, or under-informed:

After we reached here I heard nurses discussing themselves that my child is suffering from kidney cancer but they are not open with me. (Mother of 4-year-old with Wilms tumor)

A deeper communication barrier was unearthed when caregivers were asked about their child’s prognosis. All caregivers believed they were receiving curative treatment. The medical team, however, had clearly identified these children as having terminal cancers but believed it would be culturally inappropriate to directly communicate the hopeless prognosis. Because of such communication barriers, many patients had been on the oncology ward for weeks with no clear understanding of the gravity of their disease or the expected poor outcome. These prolonged hospitalizations consumed scarce medical resources—both human and material—and deepened families’ medical debt.^21^ And yet, in many instances, staff continued to proceed with life-sustaining (and often aggressive) interventions.

### Setting Advantages: Strengths and Resources

Despite these thematic challenges, reported and observational data also revealed several strengths and resources in the community and among staff, comprising a fifth and final theme. All but one caregiver reported drawing hope and/or strength from their faith, with 75% claiming a Christian background, 10% Muslim, and 15% other (Table 1). Caregivers often pointed to their faith as a source of resilience, hope, and solace and were often found praying by the bedside. Similarly, many staff also identified their faith as a source of strength in caring for terminally ill pediatric patients. Several staff also felt an ethical duty to care for patients at the end of life and thus chose to practice palliative care voluntarily—despite being employed elsewhere at BMC. For example, one nurse who worked as a surgical technician would take a *dala dala* (public taxi van) after work to visit dying patients in their homes, to ensure that they had enough pain medicines, food, and soap. Another staff member was employed as a physiotherapist but wanted badly to care for the dying and thus assembled a small, volunteer-driven Palliative Care Committee at BMC.

Another strength observed among caregivers was their deference to community cohesiveness. Decisions were not made until opinions of all key family members were heard, thereby preserving the family’s integrity. Caregivers of long-term patients were found to band together on the wards to form surrogate families and proxy communities, caring for each other’s children, sharing food, and taking turns washing diaper rags. When, for example, one mother would leave the hospital to care for children at home or gather funds, other mothers would help care for her hospitalized, terminally ill child.

## DISCUSSION

This study used a multidisciplinary qualitative approach combining interviews with participant observation for a deeper understanding of the barriers to palliative care common in a low-resource setting. Our research highlighted both previously described barriers, such as financial and infrastructure needs, and less-studied, more-complex barriers, such as cultural norms that influence clinical communication and stigmatization of death and suffering.^11,12,22,23^ The inability to discuss death presented a unique barrier to end-of-life care for children in this setting and the study thereof. For these more diffuse barriers, engaged clinical activity provided additional insight where distanced, formalized interviews might fall short. These meta-level data offered a more textured, three-dimensional appraisal of the entangled challenges at hand.^17,24,25^

Tanzania has both a national palliative care policy and a national opioid policy, stating that palliative care should be integrated into existing health care systems and that opioids such as morphine should be readily available to relieve suffering at the end of life.^12,22,26,27^ At the time of the study, however, morphine was not available at BMC, and no official positions in palliative care existed. As a result, staff under-prioritized palliative care, and cancer pain was not adequately treated. Inadequate pain management was further complicated by patients under-reporting pain, even while showing physical signs of suffering. This finding reflects sociocultural expectations of stoicism and tolerance. Cultural influences on pain expression have been described previously, although further studies are needed to create culturally relevant pain scales.^28,29^ Future research is needed to inform more feasible opioid policies in Tanzania, thereby meeting the WHO mandate to prevent and relieve suffering in all children at the end of life.^9,30^

This study also identified strengths and resources in the community that could help overcome barriers to palliative care in this context. Despite the limited treatment options and widespread stigma, BMC’s wards also hold a wealth of intangible resources. For example, BMC could draw on the spirit of collective action observed among patients and staff to increase awareness of and respect for the dying and to advocate for better access to opioids. In addition, the strong faith background found among staff and caregivers could serve as a source of comfort and resilience, which are needed to continually provide palliative care in settings such as BMC, where end-of-life needs can be overwhelming.^31,32^

There are inherent limitations to engaging in participant observation in settings such as BMC. First, conclusions drawn from a limited set of clinical experiences could be biased. Observations, analysis, and interpretation are subject to the researcher’s own background and theoretical framework.^33,34^ Second, participatory research presents ethical challenges of deciding the extent of one’s clinical involvement and when to intervene. Despite these limitations, participant observation allowed for a familiarity with context that was gained over a period of time.^17,24,25^ Even more time spent on the wards would lead to an even deeper experiential understanding.

Another limitation is that this study did not delve into the widespread use of traditional medicine for terminally ill children in Tanzania. Many children had been treated with traditional or local treatments before coming to BMC, often because of affordability and accessibility as well as a belief in spiritual dimensions of disease.^35^ Although it was outside the scope of this study, further research is needed to better understand both traditional and western medical paradigms of end-of-life care, as well as the tensions between them.

In conclusion, studies such as this that use multidisciplinary research methods provide a more holistic understanding of palliative care barriers. This study addressed the knowledge gap created by lack of cross-disciplinary communication in the study of pediatric palliative care in LMIC settings and was able to identify solutions that draw on natural strengths. Additional research using mixed, multidisciplinary methods and different ways of seeing could offer further insight and further justification for the prioritization of palliative care on the global health agenda.

Thus, further research integrating knowledge across disciplines is needed, to inform more effective policies in pediatric palliative care globally. Especially in low-resource contexts, where childhood cancers are increasing, global oncology programs face an increasing need for adequate palliative care for children.

## Appendix

### Caregiver Interview Questionnaire*

Background: Age_______, Male/female, Hometown _________________, Occupation_________________, Diagnosis_______________________________,

Hospital length of stay (days) _____

1. Why is your child hospitalized at Bugando? What will happen next in your treatment? For how long will you be here?
2. Who explained your child’s illness to you? Who updates you on the treatment plan or expected outcome?
3. Has your child had any pain during hospitalization? Did she/he receive any treatment for it? Did it help? Do you feel these treatments are safe?
  3a. [To the child]: Do you feel any pain now? (use FACES scale)
4. What are other ways you know about for treating pain? Do you, or some people you know, use local or traditional treatments for pain? What kinds? Do they help? Do you feel they are safe?
5. If your child has a problem, who do you ask for help? (Doctors, Nurses, Social workers, others) (Further probe for symptoms, payment issues, etc)
6. Do you or your child have any particular worries, concerns, or fears about your future—either in the hospital or when you go home?
7. Where do you or your child find your sources of support or hope?
8. What is your religious/spiritual background? (Probes: How involved are you? Have you become more or less involved after this illness?)
9. Once you realized your child was very sick/dying, what have you needed or wished for during hospitalization? Which of these needs were met? By whom?
10. If your neighbor or friend had a child that was seriously ill or dying and was coming to Bugando, what advice would you give them?
11. Anything else you want to share?

*Kiswahili versions available upon request

### Staff Interview Questionnaire*

Background: Age_______, Male/female, Position_________________, Years in current position____

***Axis I: Problem assessment***

Q1. How many of your pediatric patients, in a typical week, are terminally ill (defined as no further curative treatment available)?
Q1a. What do you think are the greatest needs for your dying pediatric patients?
Q1b. Are you able to address some of these needs, or not? If yes, how?

***Axis II: Structural barriers***

Q2. Are there barriers to taking adequate care of your dying pediatric patients? What are they?
Q3. In a typical day, do you feel you have enough time with each patient?
Q4. How do you identify if your patient has some serious problem, like pain or depression? Can you yourself solve this problem? Can you give an example?
Q4a. In a typical week, how often do you encounter patients who cannot afford treatments?
Q4b. Are consult services (social workers, psychologists, physical therapists, religious persons) helpful for your dying patients? How does a patient get these services? Are they usually available?

***Axis III: Barriers to pain management***

Q5. In your pediatric patients, is pain a common problem? How do you know a child is in pain?
Q6. How commonly will children get treatment for pain?
Q6a. What treatments are available? Can patients afford them?
Q6b. Are pain medications safe? Are they effective?
Q6c. What other treatments do patients use to control pain, in the hospital and at home? Are traditional medicines used? What kinds? Are traditional medicines for pain safe, and effective?

***Axis IV: Barriers to communication***

Q7. When the health care team decides a child has a terminal illness, who informs you? Who informs the patient or caretaker?
Q7a. Do most of your patients or caretakers seem to understand the child’s prognosis?
Q7b. When one of your patients is dying, do they or their families normally want to talk about it? Who do they prefer to speak with (doctors, nurses, social workers, chaplains, or other family members, etc)? Do you feel comfortable talking with them about their concerns? Did you receive any formal training on this?

***Axis VI: Barriers to education***

Q8. Are you aware of the concept of palliative care? Do you think it is important?
Q9. Have you received any training in how to care for dying patients, palliative care, or pain management? Were any of these trainings specific to the care of children?
Q9a. What sort of training in particular would be useful to you in your job?
Q9b. What gives you strength to continue caring for very sick and dying children? Do you wish you had some kind of support? If so, what?

***Additional comments (open-ended)***

Q10. Is there anything else?

*Kiswahili versions available upon request
